# Evaluation of Pre-Pectoral Direct-to-Implant Breast Reconstruction with Post-Mastectomy Radiation: A Systematic Review and Meta-Analysis

**DOI:** 10.3390/jcm14145004

**Published:** 2025-07-15

**Authors:** Nisha Parmeshwar, Jacquelyn A. Knox, Merisa L. Piper

**Affiliations:** Division of Plastic and Reconstructive Surgery, Department of Surgery, University of California San Francisco, San Francisco, CA 94143, USA; nisha.parmeshwar@ucsf.edu (N.P.); jacquelyn.knox@ucsf.edu (J.A.K.)

**Keywords:** post-mastectomy radiation therapy, direct-to-implant breast reconstruction, prepectoral breast reconstruction

## Abstract

**Background**: Immediate direct-to-implant (DTI) breast reconstruction is associated with high patient satisfaction and faster recovery. However, concerns remain for patients requiring post-mastectomy radiation therapy (PMRT). While PMRT improves overall survival for breast cancer patients, it has been associated with increased implant-specific complications such as capsular contracture, infection, and implant loss. As the impact of PMRT on pre-pectoral DTI specifically is not well understood, the goal of this systematic review was to evaluate the impact of PMRT on outcomes in this growing patient population. **Methods**: PubMed, EMBASE, and Web of Science were systematically reviewed for articles published from 1 January 2000 to 23 December 2024 investigating outcomes after prepectoral DTI reconstruction with exposure to PMRT. Demographic, clinical, and post-operative variables were recorded for PMRT and non-PMRT cohorts, and primary outcomes included infection, capsular contracture, implant loss, and wound healing complications. Meta-analysis was performed for key outcomes using the Mantel-Haenszel method. **Results**: Of 472 initially identified records, seven studies met inclusion criteria with a combined total of 343 prepectoral DTI reconstructions exposed to PMRT and 1385 reconstructions not exposed to PMRT. PMRT significantly increased the odds of any complication (OR 2.11, *p* = 0.01), implant loss (OR 1.88, *p* = 0.02), infection (OR 2.76, *p* = 0.004), and capsular contracture (OR 8.88, *p* < 0.001). However, PMRT was not associated with significantly increased odds of wound healing complications (OR 1.5, *p* = 0.36). **Conclusions**: PMRT after pre-pectoral DTI reconstruction significantly increases odds of complications, including infection, capsular contracture, and reconstructive failure. Plastic surgeons should be mindful of the sequelae of PMRT with prepectoral DTI reconstruction to improve pre-operative counseling and shared decision-making.

## 1. Introduction

The most common form of post-mastectomy reconstruction remains implant-based, encompassing 78% of all breast reconstruction procedures performed in the United States in 2023 [1]. Of these, over 36,000 procedures were performed as direct-to-implant (DTI) in a single-stage, almost three times the number of DTI procedures performed five years prior [2]. This number continues to rise each year as more surgeons and patients opt for this streamlined approach, made possible by continued advancements in mastectomy techniques, acellular dermal matrices, and prosthetic devices [3,4,5,6,7,8]. In appropriately selected patients, single-stage implant-based breast reconstruction can provide an extremely esthetic outcome with high patient satisfaction, low complication rates, and faster recovery [9,10,11].

Though post-mastectomy radiation therapy (PMRT) improves overall survival for breast cancer patients after mastectomy, it has been associated with increased complications after implant-based breast reconstruction [12,13,14,15,16,17,18]. These complications include capsular contracture, infection, need for revision surgeries, and in the most severe cases, implant loss [19]. However recent advancements in radiation therapy have improved reconstructive outcomes for patients undergoing implant-based breast reconstruction, as radiation hot spots and the volume of tissue receiving radiation are minimized in newer protocols [20]. This may also contribute to the recent rise in direct-to-implant breast reconstruction after mastectomy relative to staged and delayed procedures [21].

In light of these evolving practice patterns, still relatively few studies have commented on the impact of post-mastectomy radiation on prepectoral DTI breast reconstruction specifically. As stated, the impact of PMRT on implant-based reconstruction outcomes is well established. Yet, for most surgeons, patients selected to undergo DTI are inherently different than those selected for two-stage reconstruction [22,23,24,25]. To name a few key differences, patients selected for DTI typically have fewer comorbidities, smaller breasts, and are more likely to be assessed using indocyanine green intra-operatively—all of which play roles in both perfusion-related and general post-surgical complications. Thus, the effects of PMRT on all implant-based reconstruction patients should not be extrapolated to DTI patients without careful study. Therefore, the goal of this systematic review is to evaluate outcomes of pre-pectoral direct-to-implant breast reconstruction in patients who undergo post-mastectomy radiation therapy.

## 2. Methods

A systematic review of the literature was performed using PubMed, EMBASE, and Web of Science for articles from 1 January 2000 to 23 December 2024. Papers evaluating prepectoral direct-to-implant immediate breast reconstruction in patients exposed to post-mastectomy radiation therapy were identified. The search strategy is included in Supplemental Figure S1. Full-length articles that provided individual patient-level data, including implant plane, post-mastectomy radiation, and any of the key outcomes of interest, including infection, delayed wound healing, capsular contracture, and/or implant loss, were included. References of selected papers were also screened for additional relevant studies. Studies were excluded if they were abstracts, case reports, reviews, meta-analyses, experimental studies on animals, or primarily non-English publications. Two authors independently screened all titles and abstracts using Covidence systematic review software (Veritas Health Innovation, Melbourne, Australia). Data was extracted with a standardized collection form designed prior to study review. GRADE (Grading of Recommendations, Assessment, Development, and Evaluations) criteria was used to characterize the quality of each individual study and identify publication bias. Patient factors, including age, BMI, mastectomy indication, mastectomy type, mastectomy specimen size, implant size, and follow up time, were included when available. This review was performed in accordance with Preferred Reporting Items for Systematic Reviews and Meta-Analysis (PRISMA) guidelines.

Statistical analysis was performed using Review Manager (RevMan) Version 5.3 (Nordic Cochrane Center, the Cochrane Collaboration, Denmark). We used the Mantel-Haenszel method to calculate odds ratios (ORs) with significance level *p* < 0.05 and 95% confidence intervals for the outcomes of interest. Cumulative complication rates were pooled. A random effects model was chosen to account for interstudy heterogeneity, quantified using the I^2^ statistic.

## 3. Results

**Figure 1:**
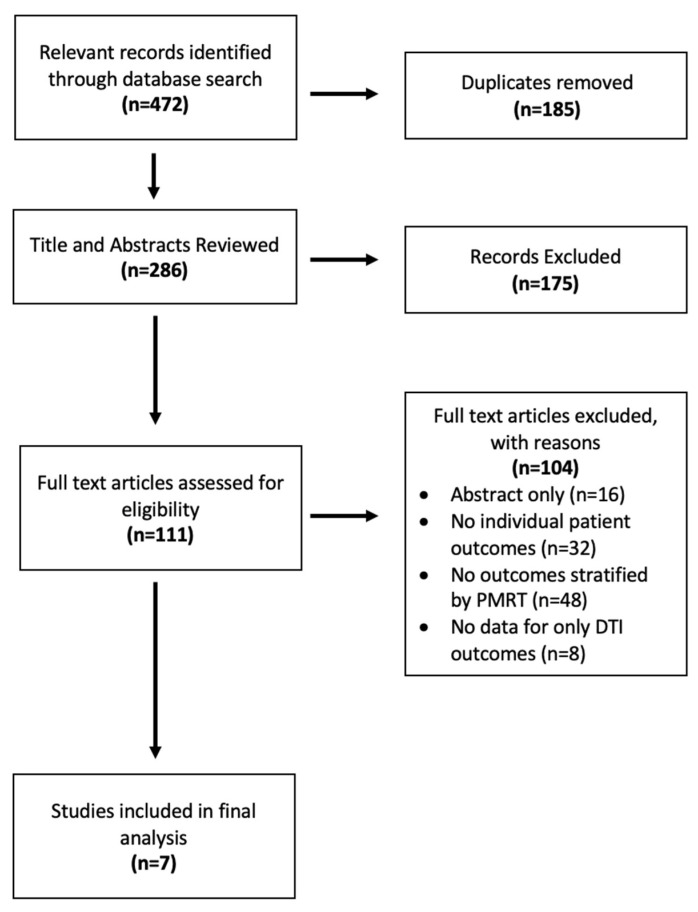
PRISMA diagram of study selection.

Of the initial 472 records identified, 185 duplicates were removed, leaving 286 distinct studies. Following title and abstract review, 175 additional studies were excluded. There were 111 studies that met full-text review screening criteria, after which only seven met our selection criteria and were included in the final analysis [26,27,28,29,30,31,32] [Figure 1]. All seven were retrospective single-institution studies, and all studies included outcomes for a PMRT cohort separate from a non-PMRT cohort and were thus included in meta-analyses. Their quality of evidence was low due to sample size and retrospective design per GRADE criteria [Table 1]. Available demographic details, including mean age, BMI, and smoking status, for each included study are shown in Table 2. Type of mastectomy, mean mastectomy weight, and/or implant size are also shown, when available, in Table 3. Mean follow up time varied from 8 months to 6 years. Across the seven studies, there were a total of 343 prepectoral DTI reconstructions exposed to post-mastectomy radiation and 1385 prepectoral DTI reconstructions that were not exposed to post-mastectomy radiation.

Included post-operative outcomes varied between studies as shown in Table 4. Six studies reported infection, capsular contracture and implant loss, so meta-analyses were conducted for these outcomes. Wound healing complications, such as skin necrosis and dehiscence, were reported for five studies, and thus included in a separate meta-analysis. While one study (Zinner et al., 2024 [32]) did not report individual complications, it did report the overall complication rate (which they defined as seroma, skin flap necrosis, scar necrosis, wound dehiscence, infection) and so was included in a meta-analysis with the six other studies where overall complication rate could be determined from available data. These complication rates were defined as all complications reported per study and, thus, varied between studies.

**Figure 2:**
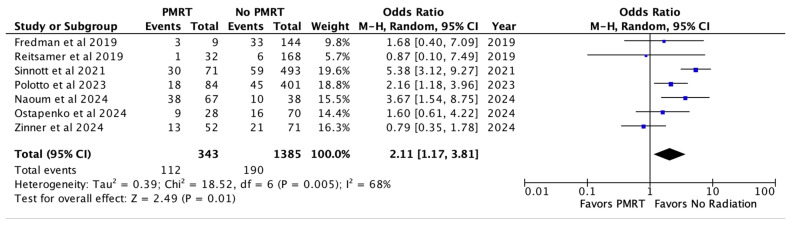
Overall complications [26,27,28,29,30,31,32].

When looking at pooled outcomes across all seven studies, the overall complication rate was 32.7% in the PMRT cohort and 13.7% in the non PMRT cohort. PMRT significantly increased the odds of any complication two-fold (OR 2.11, *p* = 0.01) [Figure 2].

**Figure 3:**
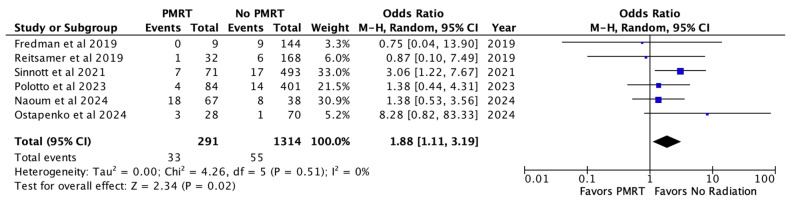
Implant loss [26,27,28,29,30,31].

Implant loss or reconstructive failure, specified across six studies, occurred in 11.3% of the PMRT cohort, and 4.1% of the non PMRT cohort. PMRT was associated with almost a two-fold increase in odds of implant loss (OR 1.88, *p* = 0.02) [Figure 3].

**Figure 4:**
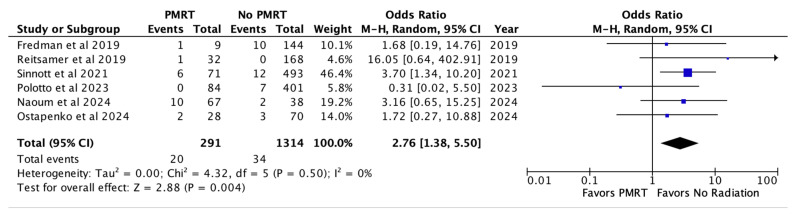
Infection [26,27,28,29,30,31].

Infection was recorded for six studies and noted in 6.8% of the PMRT and 2.6% of the non PMRT cohort. This was also statistically significant for increased odds with PMRT (OR 2.76, *p* = 0.004) [Figure 4].

**Figure 5:**
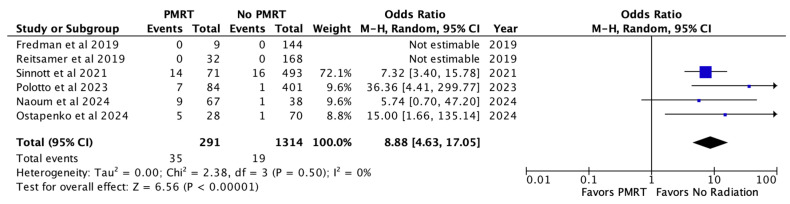
Capsular contracture [26,27,28,29,30,31].

Capsular contracture was noted in 12.0% of PMRT reconstructions and 1.4% of non-PMRT reconstructions. In meta-analysis, PMRT significantly increased the odds of capsular contracture almost nine-fold (OR 8.88, *p* < 0.00001) [Figure 5].

**Figure 6:**
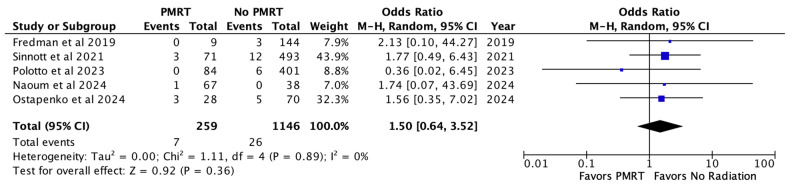
Wound healing complications [26,28,29,30,31].

Finally, wound healing complications were noted across five included studies in 2.7% of PMRT reconstructions and 2.3% of non PMRT reconstructions. There was no statistically significant increase in odds of wound healing issues with PMRT (OR 1.50, *p* = 0.36) [Figure 6].

## 4. Discussion

In this systematic review and meta-analysis, we investigated the impact of post-mastectomy radiation therapy on outcomes after single-stage pre-pectoral direct-to-implant breast reconstruction. Overall, we found that PMRT after pre-pectoral direct implant reconstruction is associated with significantly higher overall complication rates, including increased odds of infection, implant loss, and capsular contracture.

While PMRT in the setting of implant-based breast reconstruction has always been a rich area of investigation, there is no consensus on the optimal treatment strategy, in part due to the many variables at play; debate is ongoing about whether it is preferable to perform surgery in single vs. two stages, whether implants should be placed in pre- or sub-pectoral planes, and regarding the timing of radiation therapy with tissue expander placement or implant exchange. With potential benefits such as increased patient satisfaction, faster recovery, and lower post-operative pain, pre-pectoral and direct-to-implant techniques have rapidly gained popularity and are, thus, timely for more specific investigation.

Moreover, single-stage and two-stage reconstruction patients are inherently different. Chiang et al. describe numerous factors that contribute to their patient selection process, including breast size, medical comorbidities, and smoking history [25]. Furthermore, the quality of the mastectomy flap is more carefully assessed for direct-to-implant patients, either by analysis of pre-operative mammography, clinical exam, or indocyanine green angiography [5]. These confounding factors likely contribute to the heterogeneity in the current literature on whether PMRT for DTI patients does in fact lead to higher overall complication rates. For example, Abbate et al. found a lower rate of mastectomy flap necrosis in their pre-pectoral cohort compared to the sub-pectoral cohort, which contradicts previous concerns over pre-pectoral techniques [33]. This, they attribute to careful patient selection, the same of which is applied directly to implant patients. It is thus critically important to understand the potential reconstructive ramifications of PMRT for direct-to-implant patients to inform decision-making and provide thorough patient education [34].

In a previous literature review, Graziano et al. noted two-stage prepectoral implant-based breast reconstruction with PMRT should be considered safe with lower capsular contracture and implant migration rates relative to submuscular techniques [35]. Yet when Awadeen and colleagues reviewed the literature looking at the impact of PMRT on both one- and two-stage pre-pectoral implant-based breast reconstruction, they found irradiated breasts had higher rates of wound infection, capsular contracture, and implant loss [36]. This conflicting evidence regarding outcomes after radiation of prepectoral implants may be in part due to publication bias and anecdotal evidence. Some of these findings may have been attributed to the challenge of irradiating tissue expanders, which previous studies have shown may lead to more wound healing complications, capsular contracture, and reconstructive failure when compared to radiating permanent implants [37,38]. Our study adds clarity to this question by confirming that PMRT is associated with higher complication rates even without the repetitive needle accession of tissue expanders.

Furthermore, our study further challenges the heterogeneous findings of many of the individual studies included in our analysis that did look specifically at direct-to-implant reconstruction. For instance, Polotto et al. [29] concluded in their series of 84 pre-pectoral DTI patients who underwent PMRT that complication rates were overall low and acceptable, with only a 4.8% rate of implant loss. Conversely, Naoum et al. [30] reported a much higher rates of implant loss in their series of 67 pre-pec DTI patients who underwent PMRT, at 27%. As neither study provides specific details of the events leading up to the loss of reconstruction, it is difficult to explain these disparate findings. However, potential contributors include differences in patient characteristics, radiation protocols, and length of follow-up. For example, Naoum et al. [30] reported a cohort with a 30% current or former smoking rate and 75 months average follow-up, while Polotto et al. [29] reported a cohort with a 13% rate of current or former smoking and 33 months average follow-up.

Capsular contracture rates were also highly variable, with both Fredman et al. [26] and Reitsamer et al. [27] reporting 0% rates in their PMRT pre-pec DTI groups, while Sinnott et al. [28] reported an almost 20% rate of capsular contracture. This outcome is particularly challenging given the highly variable nature of measuring and reporting capsular contracture, with some studies including only Baker grades III/IV contracture while others reported only those requiring operative capsulotomy or capsulectomy, and often it is not defined either way. Furthermore, many complications, including infection, seroma, and wound complications, are not defined uniformly, and so in combining outcomes across studies, we are making assumptions regarding the similarity of how each paper chose to characterize their outcomes. Additionally, the included studies were likely underpowered to evaluate all outcomes, while others were obscured by their alternative aims, such as comparing pre- versus sub-pectoral planes. Without individual patient data, which is a limitation of systematic reviews, we cannot address these confounding variables and, thus, assume that by specifically asking the questions regarding prepectoral DTI stratified by PMRT, we have generally similar other clinical variables across groups. Finally, we cannot address differences in radiation therapy across institutions—it may be that better outcomes after radiating the permanent implant were due to differences in radiation delivery protocols and timing of radiation after mastectomy and implant placement, which are not distinguishable in this systematic review.

Future multi-institution studies are required to better elucidate the impact of such details on patient outcomes. Still, our study aids in overcoming limitations of small sample sizes at individual institutions through the increased power of systematic review and meta-analysis, demonstrating clearly increased odds of complications with PMRT in this DTI prepectoral setting, similar to prior studies looking at the prepectoral plane and two-stage reconstructions.

This study has some important limitations, including the retrospective designs of the included studies as well as the aforementioned heterogeneity in patient factors, surgical techniques, outcome measures, and radiation protocols. Certainly, factors such as patient selection, differences in mastectomy and reconstruction techniques, radiation dosing and timing, and even cancer stage or tumor biology are potential confounders we could not address. Furthermore, the substantial difference in sample size between our two groups as well as the high variability in follow-up times may introduce bias, especially considering that complications such as infection and capsular contracture can present years after reconstruction with radiation. Importantly, given the retrospective nature of the included studies, we cannot infer causality.

However, despite these limitations, our study provides novel data on the impact of PMRT on a growing population of patients undergoing direct-to-implant reconstruction. In 2017, Ricci et al. published an important meta-analysis on the timing of radiation therapy and implant-based reconstruction, concluding that regardless of timing, PMRT increases complication rates and thus autologous reconstruction should be strongly considered. However, while this study included both one and two-stage reconstructions, these groups were not isolated in their results. In 2022, Du et al. published thoughtful meta-analyses directly comparing single and two-stage implant reconstruction with and without PMRT [39]. These authors did perform a sub-meta-analysis on DTI patients but had different inclusion and search criteria as this was not their primary focus. When comparing DTI patients who received PMRT to those who did not, they found a higher rate of capsular contracture but notably did not find a higher overall complication rate. Our study hones in on direct-to-implant reconstruction patients, isolating the impact of PMRT in this group. We provide evidence with a large, combined sample size that post-mastectomy radiation therapy after prepectoral direct-to-implant breast reconstruction is associated with clinically significant increased odds of overall complications, infection, capsular contracture, and loss of reconstruction. The implications of these findings should serve both to improve patient counseling and to inform patient selection for this technique. As many patients are attracted to the single-stage nature of direct-to-implant breast reconstruction, they must be informed of these added complications with post-mastectomy radiation as to better guide their decision-making and expected post-operative sequelae.

## 5. Conclusions

Patients who undergo post-mastectomy radiation therapy after pre-pectoral direct-to-implant reconstruction have significantly increased odds of developing complications such as infection, capsular contracture, and reconstructive failure when compared to patients who did not undergo PMRT with the same reconstructive plan. Plastic surgeons offering this technique should be mindful of the sequelae related to radiating a permanent implant and set clear expectations with patients in the pre-operative period about these potential risks. Future studies are needed to investigate the outcomes of patients who need PMRT that underwent two-stage versus single-stage implant based reconstruction.

## Figures and Tables

**Table 1:** Included studies.

Year	Authors	Journal	Study Design	Years of Study	Total # Pre Pectoral DTI Recon	DTI with PMRT	DTI Without PMRT	Quality (GRADE) +
2019	Fredman et al. [26]	*Esthetic Surgery Journal*	Retrospective	2015–2016	153	9	144	Low
2019	Reitsamer et al. [27]	*The Breast*	Retrospective	2013–2018	200	32	168	Low
2021	Sinnott et al. [28]	*Annals of Plastic Surgery*	Retrospective	2010–2019	564	71	493	Low
2023	Polotto et al. [29]	*Clinical Breast Cancer*	Retrospective	2015–2020	485	84	401	Low
2024	Naoum et al. [30]	*International Journal of Radiation Oncology*	Retrospective	2005–2020	105 *	67	38	Low
2024	Ostapenko et al. [31]	*Esthetic Plastic Surgery*	Retrospective	2017–2021	98 *	28	70	Low
2024	Zinner et al. [32]	*Journal of Plastic Reconstructive Esthetic Surgery*	Retrospective	2018–2023	206	52	71	Low

* Reported patients, not breasts; + assessment of quality of evidence with GRADE (Grading of Recommendations, Assessment, Development, and Evaluations) framework; DTI = direct-to-implant; PMRT = post-mastectomy radiation therapy.

**Table 2:** Demographic variables.

Study	Total # Pre Pectoral DTI Recon	Mean Age (Years)	Mean BMI	Active Smokers
Fredman et al., 2019 [26]	153	50 (range 24–81)	26.87 (range 19.1–47.9)	3
Reitsamer et al., 2019 [27]	200	45.1 (range 25–74)	NR	NR
Sinnott et al., 2021 [28]	564	52.7 (SD 9.6)	28.7 (SD 6)	28
Polotto et al., 2023 [29]	485	54.6	23.2 (SD 3.4)	55
Naoum et al., 2024 [30]	105 *	48.3 median (IQR 42.1–57)	25.3 median (22.9–30.7 IQR)	3
Ostapenko et al., 2024 [31]	98 *	45.5 (SD 10.77)	NR	NR
Zinner et al., 2024 [32]	206	45.7 (SD 39.4)	24.5 (SD 4.3)	30

* Reported patients, not breasts; NR = Not recorded.

**Table 3:** Clinical and surgical data.

Study	Total # Pre Pectoral DTI Recon	Mastectomy Type	Mastectomy Indication	Mean Mastectomy Specimen Weight (g)	Mean Implant Size (cc)	Mean Follow-Up Time (Months)
Fredman et al., 2019 [26]	153	92 NSM 61 SSM	70 prophylactic 83 cancer	622.66 g ± 401.3	586.16 ± 157.76	8.5 ± 3.9 (range 3–17)
Reitsamer et al., 2019 [27]	200	200 NSM	51 prophylactic 149 cancer	NR	340 (range 110–735)	36 (3–68 range)
Sinnott et al., 2021 [28]	564	NR	NR	NR	370.2 ± 120.6	18.3 ± 17.7
Polotto et al., 2023 [29]	485	433 NSM 52 SSM	NR	NR	No PMRT: 383 ± 110 PMRT: 399 ± 106	No PMRT: 32.4 ± 17.8 PMRT: 33.3 ± 15.8 Range: 8.3–84
Naoum et al., 2024 [30]	105 *	NR	NR	NR	NR	6.2 years (IQR 0.7–16.4)
Ostapenko et al., 2024 [31]	98 *	98 NSM	12 prophylactic 86 therapeutic	NR	364.3 cc ± 78.1	31.12 ± 14.5
Zinner et al., 2024 [32]	206	NSM 74 70 SSM	123 patients with breast cancer	410.9 g ± 263.7	NR	NR

* Reported patients, not breasts; NR = not recorded; NSM = nipple sparing mastectomy; SSM = skin sparing mastectomy.

**Table 4:** Individual study outcomes.

Study	Any Complication		Implant Loss/Reconstructive Failure		Infection		Capsular Contracture		Wound Healing		Other	
	PMRT	None	PMRT	None	PMRT	None	PMRT	None	PMRT	None	PMRT	None
Fredman et al., 2019 [26]	3/9	33/144	0/9	9/144	1/9	10/144	0/9	0/144	0/9	3/144	1 Implant replaced 0 seroma	0 implants replaced 4 seromas
Reitsamer et al., 2019 [27]	1/32	6/168	1/32	6/168	1/32	0/168	0/32	0/168				
Sinnott et al., 2021 [28]	30/71	59/493	7/71	17/493	6/71	12/493	14/71	16/493	3/71	12/493	0 hematoma 0 seroma	1 hematoma 1 seroma
Polotto et al., 2023 [29]	18/84	45/401	4/84	14/401	0/84	7/401	7/84	1/401	0/84	6/401	0 hematoma 6 seromas	1 hematoma 15 seromas
Naoum et al., 2024 [30]	38/67	10/38	18/67	8/38	10/67	2/38	9/67	1/38	1/67	0/38		
Ostapenko et al., 2024 [31]	9/28	16/70	3/28	1/70	2/28	3/70	5/28	1/70	3/28	5/70		
Zinner et al., 2024 [32]	13/52	21/71	Any complications defined as seroma skin flap necrosis, scar necrosis, wound dehiscence, infection for this study									

## Supplementary Materials

The following supporting information can be downloaded at: https://www.mdpi.com/article/10.3390/jcm14145004/s1, Figure S1: Search strategy. Figure S2 Overall Complications. Figure S3: Implant Loss. Figure S4: Infection. Figure S5: Capsular Contracture. Figure S6: Wound Healing Complications. Table S1: Included Studies. Table S2: Demographic Variables. Table S3: Clinical and Surgical Data Per Study. Table S4: Individual Study Outcomes. PRISMA 2020 checklist.
