# Supplementary material for: Evaluation of Pre-Pectoral Direct-to-Implant Breast Reconstruction with Post-Mastectomy Radiation: A Systematic Review and Meta-Analysis
Source: J Clin Med. 2025 Jul 15;14(14):5004. doi: 10.3390/jcm14145004 (PMC12295042; doi:10.3390/jcm14145004)
Supplement: Supplementary file 1 [file jcm-14-05004-s001.zip › jcm-3643509-supplementary.pdf]

Table S1 Included Studies

Year	Authors	Journal	Study Design	Years of Study	Total # Pre Pectoral DTI Recon	DTI with PMRT	DTI without PMRT	Quality (GRADE)+
2019	Fredman et al	*Aesthetic Surgery Journal*	Retrospective	2015-2016	153	9	144	Low
2019	Reitsamer et al	*The Breast*	Retrospective	2013-2018	200	32	168	Low
2021	Sinnott et al	*Annals of Plastic Surgery*	Retrospective	2010-2019	564	71	493	Low
2023	Polotto et al	*Clinical Breast Cancer*	Retrospective	2015-2020	485	84	401	Low
2024	Naoum et al	*International Journal of Radiation Oncology*	Retrospective	2005-2020	105*	67	38	Low
2024	Ostapenko et al	*Aesthetic Plastic Surgery*	Retrospective	2017-2021	98*	28	70	Low
2024	Zinner et al	*Journal of Plastic Reconstructive Aesthetic Surgery*	Retrospective	2018-2023	206	52	71	Low

*reported patients, not breasts
+assessment of quality of evidence with GRADE (Grading of Recommendations, Assessment, Development, and Evaluations) framework
DTI= direct to implant
PMRT= post-mastectomy radiation therapy

Table S2 Demographic Variables

Year	Authors	Total # Pre Pectoral DTI Recon	Mean Age (years)	Mean BMI	Active Smokers
2019	Fredman et al	153	50 (range 24-81)	26.87 (range 19.11-47.91)	3
2019	Reitsamer et al	200	45.1 (25-74)	NR	NR
2021	Sinnott et al	564	52.7 (SD 9.6)	28.7 (SD 6)	28
2023	Polotto et al	485	54.55	23.2 (SD 3.4)	55
2024	Naoum et al	105*	48.3 median (IQR 42.1-57)	25.3 median (22.9-30.7 IQR)	3
2024	Ostapenko et al	98*	45.5 (SD 10.77)	NR	NR
2024	Zinner et al	206	45.7 (SD 39.4)	24.48 (SD 4.3)	30

*reported patients, not breasts
NR= Not recorded

Table S3 Clinical and Surgical Data Per Study

Year	Authors	Total # Pre Pectoral DTI Recon	Mastectomy type	Mastectomy indication	Mean Mastectomy Specimen Weight (g)	Mean Implant Size (cc)	Mean follow up time
2019	Fredman et al	153	92 NSM<br>61 SSM	70 prophylactic<br>83 cancer	622.66g ± 401.3	586.16 ± 157.76	8.5 months ± 3.9 (range 3-17)
2019	Reitsamer et al	200	200 NSM	51 prophylactic<br>149 cancer	NR	340 (range 110-735)	36 months (3-68 range)
2021	Sinnott et al	564	NR	NR	NR	370.2 ± 120.6	18.3 months ± 17.7
2023	Polotto et al	485	433 NSM<br>52 SSM	NR	NR	No PMRT: 383 ± 110<br>PMRT: 399 ± 106	No PMRT: 32.4 ± 17.8 months<br>PMRT: 33.3 ± 15.8 months<br><br>Range: 8.3-84 months
2024	Naoum et al	105*	NR	NR	NR	NR	6.2 years (IQR 0.7-16.4)
2024	Ostapenko et al	98*	98 NSM	12 prophylactic<br>86 therapeutic	NR	364.3cc ± 78.1	31.12 ± 14.5 months
2024	Zinner et al	206	NSM 74<br>70 SSM	123 patients with breast cancer	410.9g ± 263.7	NR	NR

*reported patients, not breasts
NR= Not recorded
NSM= nipple sparing mastectomy
SSM= skin sparing mastectomy

Table S4 Individual Study Outcomes

Study	Any Complication		Implant Loss/ Reconstructive Failure		Infection		Capsular Contracture		Wound Healing		Other	
	PMRT	None	PMRT	None	PMRT	None	PMRT	None	PMRT	None	PMRT	None
**Fredman et al 2019**	3/9	33/144	0/9	9/144	1/9	10/144	0/9	0/144	0/9	3/144	1 Implant replaced<br>0 Seroma	0 implants replaced<br>4 Seroma
**Reitsamer et al 2019**	1/32	6/168	1/32	6/168	1/32	0/168	0/32	0/168				
**Sinnott et al 2021**	30/71	59/493	7/71	17/493	6/71	12/493	14/71	16/493	3/71	12/493	0 hematoma<br>0 seroma	1 hematoma<br>1 seroma
**Polotto et al 2023**	18/84	45/401	4/84	14/401	0/84	7/401	7/84	1/401	0/84	6/401	0 hematoma<br>6 seroma	1 hematoma<br>15 seroma
**Naoum et al 2024**	38/67	10/38	18/67	8/38	10/67	2/38	9/67	1/38	1/67	0/38		
**Ostapenko et al 2024**	9/28	16/70	3/28	1/70	2/28	3/70	5/28	1/70	3/28	5/70		
**Zinner et al 2024**	13/52	21/71	*Any complications defined as seroma skin flap necrosis, scar necrosis, wound dehiscence, infection for this study									

Figure S1 PRISMA Diagram of Study Selection

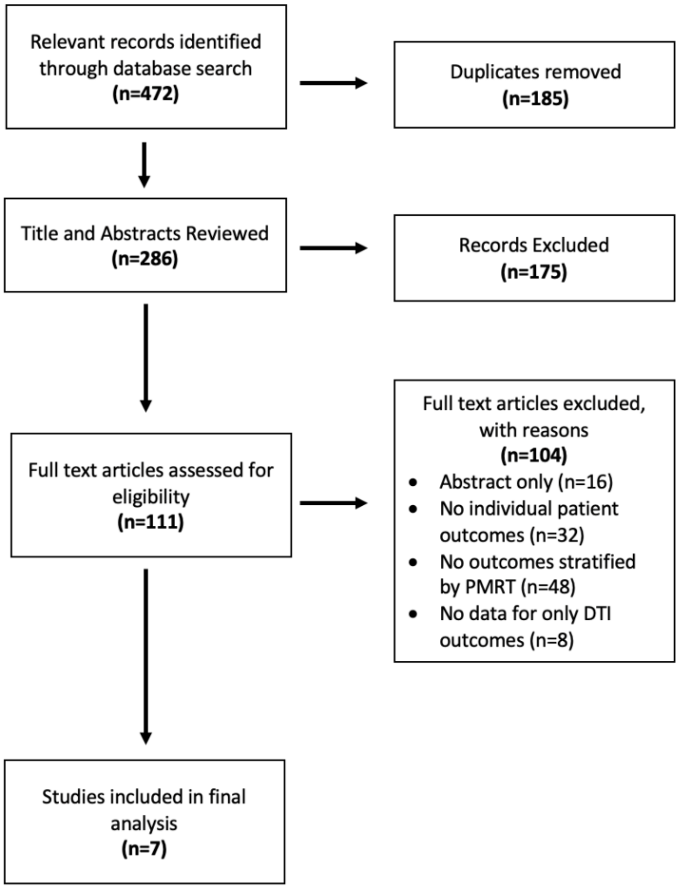

Figure S2 Overall Complications

Study or Subgroup	PMRT Events	PMRT Total	No PMRT Events	No PMRT Total	Weight	Odds Ratio M-H, Random, 95% CI	Year
Fredman et al 2019	3	9	33	144	9.8%	1.68 [0.40, 7.09]	2019
Reitsamer et al 2019	1	32	6	168	5.7%	0.87 [0.10, 7.49]	2019
Sinnott et al 2021	30	71	59	493	19.6%	5.38 [3.12, 9.27]	2021
Polotto et al 2023	18	84	45	401	18.8%	2.16 [1.18, 3.96]	2023
Naoum et al 2024	38	67	10	38	15.5%	3.67 [1.54, 8.75]	2024
Ostapenko et al 2024	9	28	16	70	14.4%	1.60 [0.61, 4.22]	2024
Zinner et al 2024	13	52	21	71	16.3%	0.79 [0.35, 1.78]	2024
**Total (95% CI)**		**343**		**1385**	**100.0%**	**2.11 [1.17, 3.81]**	
Total events	112		190				

Heterogeneity: $Tau^2 = 0.39$; $Chi^2 = 18.52$, df = 6 (P = 0.005); $I^2 = 68\%$
Test for overall effect: Z = 2.49 (P = 0.01)

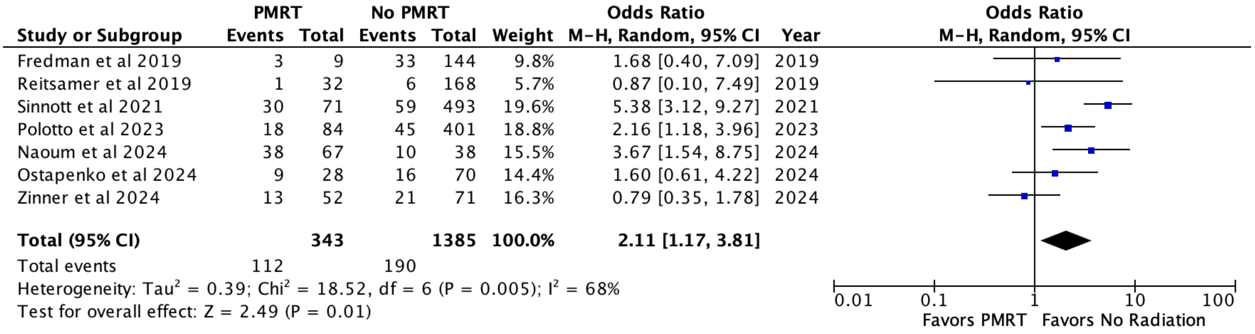

Figure S3: Implant Loss

Study or Subgroup	PMRT Events	PMRT Total	No PMRT Events	No PMRT Total	Weight	Odds Ratio M-H, Random, 95% CI	Year
Fredman et al 2019	0	9	9	144	3.3%	0.75 [0.04, 13.90]	2019
Reitsamer et al 2019	1	32	6	168	6.0%	0.87 [0.10, 7.49]	2019
Sinnott et al 2021	7	71	17	493	33.0%	3.06 [1.22, 7.67]	2021
Polotto et al 2023	4	84	14	401	21.5%	1.38 [0.44, 4.31]	2023
Naoum et al 2024	18	67	8	38	30.9%	1.38 [0.53, 3.56]	2024
Ostapenko et al 2024	3	28	1	70	5.2%	8.28 [0.82, 83.33]	2024
**Total (95% CI)**		**291**		**1314**	**100.0%**	**1.88 [1.11, 3.19]**	
Total events	33		55				

Heterogeneity: $Tau^2 = 0.00; Chi^2 = 4.26, df = 5 (P = 0.51); I^2 = 0\%$
Test for overall effect: Z = 2.34 (P = 0.02)

Odds Ratio
M-H, Random, 95% CI

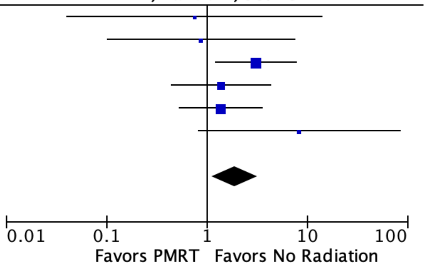

Figure S4: Infection

Study or Subgroup	PMRT Events	PMRT Total	No PMRT Events	No PMRT Total	Weight	Odds Ratio M-H, Random, 95% CI	Year
Fredman et al 2019	1	9	10	144	10.1%	1.68 [0.19, 14.76]	2019
Reitsamer et al 2019	1	32	0	168	4.6%	16.05 [0.64, 402.91]	2019
Sinnott et al 2021	6	71	12	493	46.4%	3.70 [1.34, 10.20]	2021
Polotto et al 2023	0	84	7	401	5.8%	0.31 [0.02, 5.50]	2023
Naoum et al 2024	10	67	2	38	19.2%	3.16 [0.65, 15.25]	2024
Ostapenko et al 2024	2	28	3	70	14.0%	1.72 [0.27, 10.88]	2024
**Total (95% CI)**		**291**		**1314**	**100.0%**	**2.76 [1.38, 5.50]**	
Total events	20		34				

Heterogeneity: $Tau^2 = 0.00; Chi^2 = 4.32, df = 5 (P = 0.50); I^2 = 0\%$
Test for overall effect: Z = 2.88 (P = 0.004)

Odds Ratio
M-H, Random, 95% CI

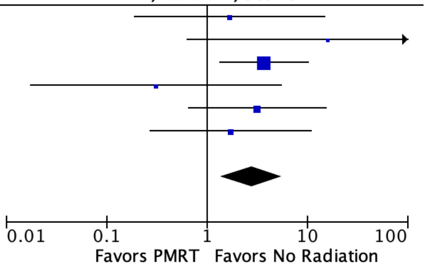

Figure S5: Capsular Contracture

Study or Subgroup	PMRT Events	PMRT Total	No PMRT Events	No PMRT Total	Weight	Odds Ratio M-H, Random, 95% CI	Year
Fredman et al 2019	0	9	0	144		Not estimable	2019
Reitsamer et al 2019	0	32	0	168		Not estimable	2019
Sinnott et al 2021	14	71	16	493	72.1%	7.32 [3.40, 15.78]	2021
Polotto et al 2023	7	84	1	401	9.6%	36.36 [4.41, 299.77]	2023
Naoum et al 2024	9	67	1	38	9.6%	5.74 [0.70, 47.20]	2024
Ostapenko et al 2024	5	28	1	70	8.8%	15.00 [1.66, 135.14]	2024
**Total (95% CI)**		**291**		**1314**	**100.0%**	**8.88 [4.63, 17.05]**	
Total events	35		19				

Heterogeneity: $Tau^2 = 0.00; Chi^2 = 2.38, df = 3 (P = 0.50); I^2 = 0\%$
Test for overall effect: Z = 6.56 (P < 0.00001)

Odds Ratio
M-H, Random, 95% CI

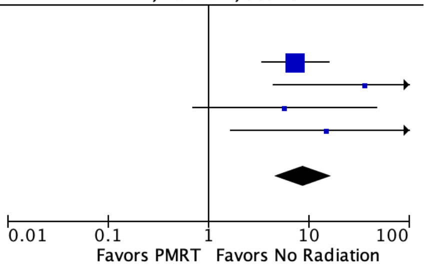

Figure S6: Wound Healing Complications

Study or Subgroup	PMRT Events	PMRT Total	No PMRT Events	No PMRT Total	Weight	Odds Ratio M-H, Random, 95% CI	Year
Fredman et al 2019	0	9	3	144	7.9%	2.13 [0.10, 44.27]	2019
Sinnott et al 2021	3	71	12	493	43.9%	1.77 [0.49, 6.43]	2021
Polotto et al 2023	0	84	6	401	8.8%	0.36 [0.02, 6.45]	2023
Naoum et al 2024	1	67	0	38	7.0%	1.74 [0.07, 43.69]	2024
Ostapenko et al 2024	3	28	5	70	32.3%	1.56 [0.35, 7.02]	2024
**Total (95% CI)**		**259**		**1146**	**100.0%**	**1.50 [0.64, 3.52]**	
Total events	7		26				

Heterogeneity: $Tau^2 = 0.00; Chi^2 = 1.11, df = 4 (P = 0.89); I^2 = 0\%$
Test for overall effect: Z = 0.92 (P = 0.36)

Odds Ratio
M-H, Random, 95% CI

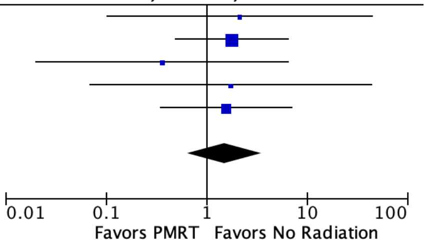

Supplemental Figure 1: Search Strategy

Prepectoral implants and radiation therapy
Search run: 12/23/2024

PubMed

("prepectoral"[tw] OR "pre pectoral"[tw]) AND ("radiotherapy"[MeSH Subheading] OR "radiotherapy"[tw] OR "radiation therapy"[tw] OR "radiotherapy"[MeSH Terms]) NOT ("systematic review"[pt] OR "Meta-Analysis"[pt])

Filters: NOT systematic review/meta-analysis (already integrated into search)
Results: 105

Embase

('prepectoral':ti,ab,kw,de,dn,df,mn,tn OR 'pre pectoral':ti,ab,kw,de,dn,df,mn,tn) AND ('radiotherapy':ti,ab,kw,de,dn,df,mn,tn OR 'radiation therapy':ti,ab,kw,de,dn,df,mn,tn OR 'radiotherapy'/exp) NOT ('systematic review':it OR 'meta-analysis':it)

Filters: NOT systematic review/meta-analysis (already integrated into search)
Results: 212

Web of Science

("prepectoral" OR "pre pectoral") AND ("radiotherapy" OR "radiation therapy")

Filters: Topic Search
Results: 152
